# Identification and Abiotic Stress Expression Profiling of Malic Enzyme-Associated Genes in Maize (*Zea mays* L.)

**DOI:** 10.3390/plants14111603

**Published:** 2025-05-24

**Authors:** Haishan Yan, Yongsheng Li, Zengke Ma, Ruihong Wang, Yuqian Zhou, Wenqi Zhou, Haijun He, Xiaojuan Wang, Xiaorong Lian, Xiaoyun Dong, Lirong Yao

**Affiliations:** 1 College of Agriculture, Gansu Agricultural University, Lanzhou 730070, China; 18846301250@163.com (H.Y.); 17339902200@163.com (R.W.); 2 Crop Research Institute, Gansu Academy of Agricultural Sciences, Lanzhou 730070, China; zengkema@sina.com (Z.M.); zhouyuqian@gsagr.cn (Y.Z.); zhouwenqi850202@163.com (W.Z.); hhj007@sina.com (H.H.); wangxj839@sina.com (X.W.); lianxr@126.com (X.L.); dongxy@st.gsau.edu.cn (X.D.)

**Keywords:** maize, malic enzyme, gene family identification, gene expression

## Abstract

Malic enzyme (ME), a key enzyme involved in various metabolic pathways, catalyzes the oxidative decarboxylation of malate to generate pyruvate, CO_2_, and NADPH. This enzyme plays essential roles in plant growth, development, and stress responses. In this study, 13 maize *ME* genes were identified by performing homologous sequence alignment using the sequences of the *Arabidopsis ME* gene family as references. Chromosomal localization analysis demonstrated that *ME* genes were not detected on chromosomes 9 and 10, whereas the remaining eight chromosomes exhibited an uneven distribution of these genes. Phylogenetic analysis indicated a high degree of conservation between maize *ME* genes and their orthologs in teosinte (*Zea luxurians* L.) throughout the evolutionary history of Poaceae crops. Furthermore, cis-acting element analysis of promoters demonstrated that members of the maize *ME* gene family harbor regulatory elements associated with stress responses, phytohormones signaling, and light responsiveness, which suggests their potential role in abiotic stress adaptation. Expression profiling under stress conditions revealed differential expression levels of maize *ME* genes, with *ZmME13* emerging as a promising candidate gene for enhancing stress resistance. These results lay a solid foundation for further investigation into the biological functions of the maize *ME* gene family.

## 1. Introduction

Maize (*Zea mays* L.) is the most widely cultivated food crop worldwide, and it is also an important source of animal feed and raw materials for industrial processing [1]. During the growth of maize, high temperatures, droughts, and salty soils can significantly impact its yields [2]. Drought, considered the most significant abiotic stress factor, would hinder maize’s normal growth and development by disrupting its normal physiological processes, ultimately resulting in severe yield losses [3]. The global decrease in maize yield due to drought is about 15% each year [4]. Prolonged drought directly interferes with various physiological indicators of maize, forcing the acceleration of various physiological and biochemical reactions in the maize reproductive process, shortening the maize growth cycle, resulting in significantly smaller cobs and reduced dry matter accumulation, and ultimately leading to a significant decline in maize quality and yield. In extreme cases, it can even lead to a significant reduction in maize yields as well as crop failure [5,6]. At present, countries around the world seek solutions to their dilemmas through research on dry farming, water-saving agriculture, drought-resistant breeding, and the traditional genetic breeding technology of maize. Although drought-resistant maize improvement has played a role, the cultivation of new drought-resistant maize varieties, is needed to realize an effective solution to the problem. For high and stable yields of maize, it is necessary to select and breed drought-resistant and high-temperature-resistant maize materials. Research on drought and heat tolerant maize varieties should aim to understand the molecular mechanisms of its drought and heat tolerance, particularly focusing on the synergistic regulation of stress-responsive genes and epigenetic adaptations during the entire maize reproductive period. The flowering, pollen dispersal, and grouting periods are the most vulnerable to drought and high temperatures [7], during which high temperatures can lead to decreased pollen viability or abortion, shriveling of filaments, and reduced kernel fullness and quality. Under high temperatures and drought stress, stress response phenomena occur in plants, and there are many genes whose expression is repressed or stimulated, i.e., up-regulated or down-regulated [8,9]. Given the significant impact of drought on maize, understanding the underlying physiological and molecular mechanisms is crucial. Malic acid and its related enzymes, such as malic enzyme, have been shown to be involved in plant stress responses [10,11] which provides a potential research direction for exploring maize drought tolerance.

Malic acid (MA) plays an important function in plant growth and development [12,13]. In plant cells, malic acid is a substance that links multiple metabolisms in different organelles and is involved in the regulation of a variety of metabolic reactions. Through malate synthesis and oxidation reactions, cells function as reducing power carriers and carbon carriers. The enzymes that catalyze the synthesis and oxidation of malic acid are malate synthase, malic enzyme and malate dehydrogenase [14]. Based on the coenzyme factors, plant malic enzymes can be divided into NAD-dependent malic enzymes and NADP-ME [15]. Malic enzyme is an important precursor for producing ketone bodies [16]. Studies have shown that plant malic enzymes not only play a significant role in key metabolic pathways such as photosynthesis and respiration, but also regulate plant growth and development [17], potentially related to plant defense mechanisms [11,18]. Ice plum contains the *NADP-ME* gene [19], which is induced under NaCl stressed conditions. The activities of both NAD-ME and NADP-ME in the plant also increase under stress [20]. Rice (*Oryza sativa* L.) has two *NADP-ME* genes that are induced under NaCl, NaHCO_3_, and Na_2_CO_3_ stressed conditions, with their activities accordingly increasing. Overexpression of these genes in transgenic *Arabidopsis thaliana* increases the plant’s salt tolerance [21,22]. Under drought conditions, the expression of *NADP-ME* genes is also affected by drought stress, but their response patterns differ [23,24,25]. Plants are exposed to seasonal temperature variations that significantly affect their biochemical processes. Elevated temperatures can cause the thermal deactivation of enzymes, resulting in the structural disassembly of enzymatic systems and cellular dehydration. In contrast, suboptimal low temperatures decrease root vitality, protoplasmic fluidity, and NADP-ME activity, while concurrently inducing chlorophyll degradation via photooxidative mechanisms. These temperature-induced disturbances collectively disrupt metabolic homeostasis in plants [26]. NADP-ME in wheat leaf increases gradually under 4 in 24 h. However, the index of photosynthesis does not show the same trend. This means that the activity of NADP-ME seriously affected by low temperatures. The enzyme responds to stress at the protein level. Through RT-PCR study, *TaNADP-ME1* and *TaNADP-ME2* showed the same variation trend, which increased after 3 h of declining under low-temperature stress. This evidence indicates that cold stress can induce the expression of the *NADP-ME* gene, which has a close relationship with the cold resistance of plants [27].

In this study, the maize inbred line B73 was selected as the research subject. Bioinformatics approaches were employed to systematically analyze the physicochemical properties, phylogenetic relationships, chromosomal localization and collinearity of the ME gene family. These extensive data resources establish a robust theoretical foundation and provide critical support for further exploration of the functional roles of drought tolerance-associated genes within the *ME* gene family in maize. Through this integrated methodology, the research aims to deepen the understanding of plant drought resistance mechanisms, identify novel molecular targets for stress adaptation, and ultimately drive revolutionary advancements in crop improvement and agricultural productivity.

## 2. Results

### 2.1. Identification of the Maize ME Gene Family

Based on sequence information from Uniport for six MEs (NADP-ME and NAD-ME) in *Arabidopsis thaliana*, a bioinformatics analysis was performed to study their protein sequence characteristics. BLAST searches were conducted to identify *ZmME* gene family members in the protein sequences of maize, teosinte (*Zea* spp.), sorghum (*Sorghum bicolor* L.), rice (*Oryza sativa* L.), barley (*Hordeum vulgare* L.), millet (*Seteria italica* L.), and proso millet (*Panicum meliaceous* L.). Using the local BLASTP v.2.6.0 tool (E < 1 × 10^−10^, identify > 50%), 13 *ZmME* gene family members were identified and analyzed as *ZmME1~ZmME13* from the maize genome (Table 1). The sequence properties of these members indicate a range of lengths from 1034 to 54,178 bp for the genes and 414 to 1932 codons for the coding regions, corresponding to protein molecular weights ranging from 15,622.11 kDa to 70,694.85 kDa and amino acid counts of 178 to 644. *ZmME5* contains only 178 amino acids (the shortest), while *ZmME10* is the longest at 644 amino acids. The theoretical isoelectric point (pI) ranges from 5.22 to 9.2, with *ZmME1*, *ZmME5*, *ZmME7*, and *ZmME8* having pI values above 7 (basic proteins), while the remaining members are acidic proteins. The overall hydrophobicity coefficient spans a range of −0.361~0.291, the average hydrophilicity coefficient values are consistently less than zero, indicating kinetically favorable properties for these proteins to bind water.

### 2.2. Chromosomal Localization and Collinearity Analysis

**Figure 1:**
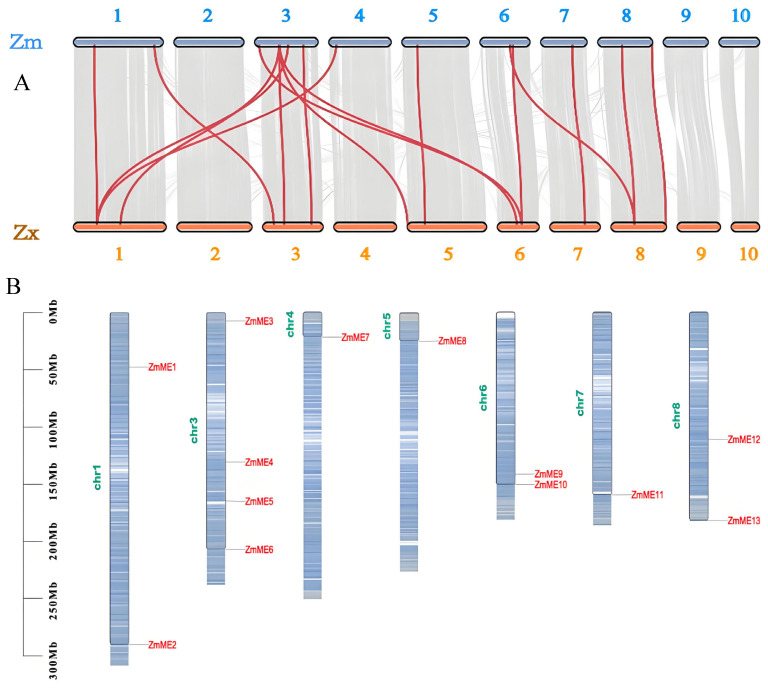
Chromosomal localization and collinearity analysis. (**A**) Represents the collinearity between Zm and Zx; (**B**) Represents chromosomal localization.

The collinearity analysis results demonstrated that *ME* genes were not detected on chromosomes 9 and 10, whereas the remaining eight chromosomes exhibited an uneven distribution of these genes (Figure 1A). Among these chromosomes, chromosome 3 contained four *ZmME* genes, while the others carried one to two *ZmME* genes each (Figure 1B). This suggests that *ZmME* gene family members are unevenly dispersed across chromosomes. Additionally, 16 collinear relationships were identified between the 13 *ZmME* genes and 12 *ZxME* genes (Figure 1A). Comparative analysis indicated that *ZmME* genes and Zx*ME* genes exhibited minimal accumulated variations, sharing numerous conserved features and demonstrating high evolutionary conservation.

### 2.3. Phylogenetic Analysis of Gene Families

**Figure 2:**
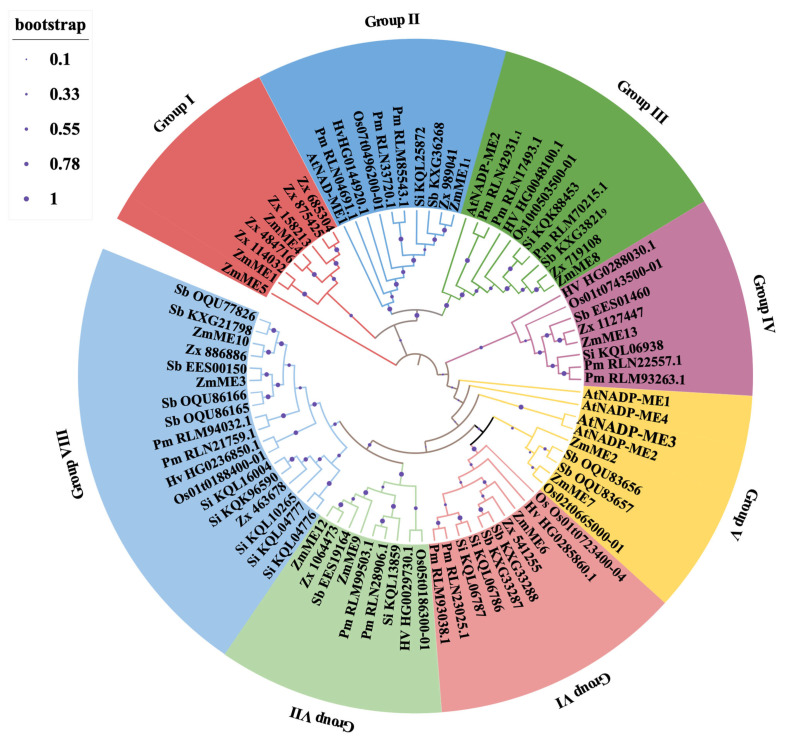
Developmental trees of maize, teosinte, sorghum, rice, barley, millet, proso millet and arabidopsis phylogenetic tree.

The results revealed that the 82 ME proteins were classified into eight subfamilies, with an uneven distribution of *ZmME* genes across these subfamilies (Figure 2). Subfamily I, the smallest group comprising only eight members, harbored the highest number of *ZmME* genes (three). In contrast, subfamilies II, III, IV, and VI each contained a single *ZmME* gene, whereas subfamilies V, VII, and VIII exhibited two *ZmME* genes per subfamily. Notably, phylogenetic analysis demonstrated that maize ME proteins exhibited the closest evolutionary relationship to and highest homology with those of teosinte ME suggesting functional conservation between maize and teosinte.

### 2.4. Conserved Motifs, Structural Domains and Gene Structure Analysis of Gene Family Members

**Figure 3:**
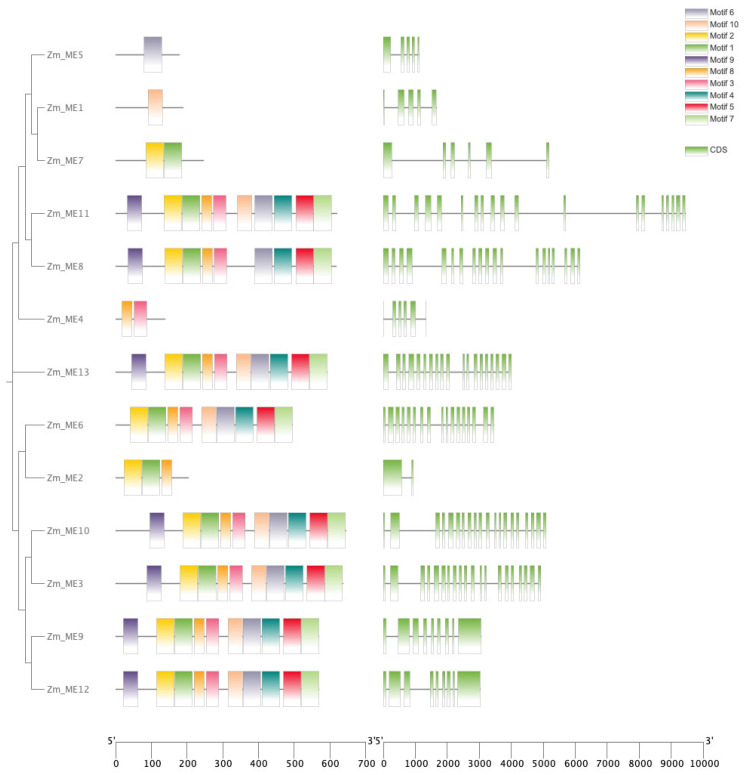
Protein conserved motifs and gene structure analysis of *ZmME*.

An analysis of the systematics of *ZmME* gene family members revealed (Figure 3) that 13 *ZmME* genes exhibited 1–10 conserved motifs, and these 13 *ZmME* genes were divided into three major branches. In the first branch, *ZmME5* contained only one conserved motif Motif6, while *ZmME1* contained only one conserved motif Motif10. The second branch included *ZmME7* with two conserved motifs Motif2 and Motif1 and *ZmME4* with two conserved motifs Motif8 and Motif3. In the third branch, *ZmME2* had three conserved motif Motif2, Motif1, and Motif8, while the other developmental groups exhibited high similarity in their conserved motif. Conserved motifs are typically associated with protein functions. These motifs’ sequence information was submitted to the PFAM database for functional queries. The results demonstrated that the majority of genes were associated with oxidoreductase activity (GO:0016616) and exhibited malic enzyme activity (GO:0004470), indicating that these motifs play a pivotal role in the catalytic process of malic enzyme generation. The results indicate that the predicted conserved motif within the same developmental group of the *ZmME* gene family exhibit a high degree of identity and share relatively similar genetic structures.

### 2.5. Analysis of Promoter Cis-Acting Elements of Maize ME Family Members

**Figure 4:**
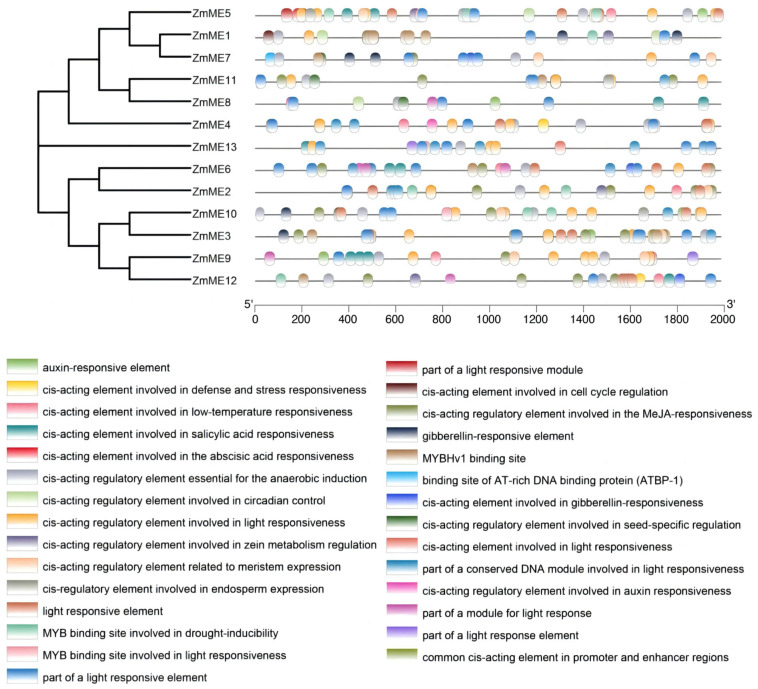
Distribution of cis-acting elements in the initiator region of *ZmME*.

The bioinformatic analysis of cis-regulatory elements in the 13 ZmME genes identified 29 functionally significant elements within this gene family. (Figure 4). In maize, the promoters of these 13 *ZmME* genes contain varying numbers of functional elements such as growth hormone response elements TGA-box, GARE, and GCC-box, TGA-box regulates the expression of antioxidant enzyme genes (e.g., glutathione transferase) to mitigate oxidative damage. GARE controls stem elongation-related genes (e.g., GA20ox), promoting internode growth and flowering. GCC-box enhances stress resilience by enabling EIN3 binding under hypoxia or salt stress to activate stress-responsive genes. Integration of TGA-box and GCC-box coordinates plant adaptation to multiple stressors, abscisic acid trans-acting elements, and light response elements. Additionally, apart from the basic promoter elements such as CAAT boxes and TAAT boxes, they also possess multiple stress-related responsive elements, including ABA response elements, defense and stress response elements, heat response elements, cold response elements, and motifs associated with abiotic stress-induced responses. Furthermore, these promoters exhibit binding sites specifically related to drought-induction processes. These findings suggest that the *ZmME* gene family may play significant roles in developmental processes, such as regulating seed maturation and storage reserve accumulation, root elongation and morphogenesis, and reproductive growth, further indicating the critical regulatory functions of *ME* genes under adverse environmental conditions.

### 2.6. Expression Analysis of Maize Malic Enzyme Gene Under Abiotic Stresses

To investigate the expression patterns of the *ZmME* gene family under various abiotic stress conditions, this study examined the expression levels of different *ZmME* genes in seedlings at 42 °C high-temperature stress, 4 °C low-temperature stress, 250 mmol NaCl salinity stress, 20% PEG polyethylene glycol stress, and drought treatments at time points of 0 h (CK), 1 h, 3 h, 6 h, 12 h, and 24 h. Table 2 Primer information used in the transcriptional expression level detection of ZmMEgenes. The results showed that all five types of abiotic stress induced distinct differential effects on the expression of *ZmME* genes.

#### 2.6.1. Drought Stress Treatments

**Figure 5:**
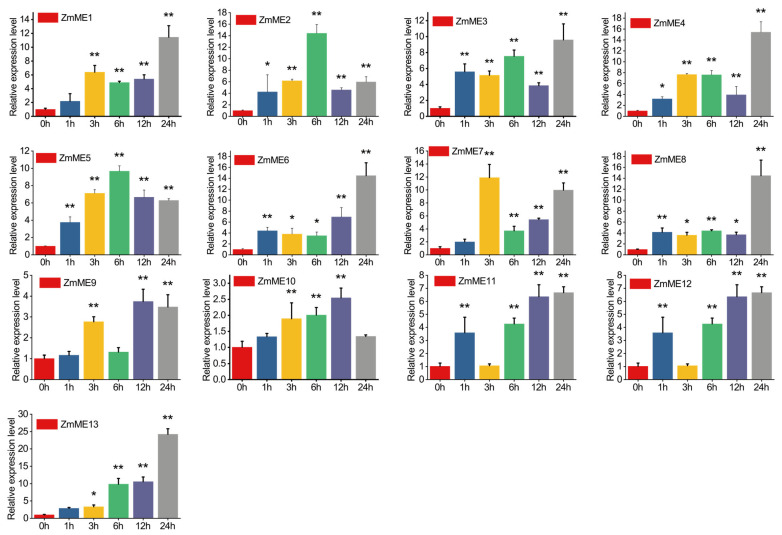
Induced expression results of *ZmME* under drought stress conditions. (*) indicates a significant difference; (**) denotes a highly significant difference. Red represents 0 h (ck), blue represents the expression level at 1 h, yellow at 3 h, green at 6 h, purple at 12 h, and grey at 24 h.

To investigate the drought responsiveness of the *ZmME* gene family, we evaluated the expression profiles of *ZmME* genes in maize leaf tissue under drought stress (Figure 5, Table S1). Thirteen *ZmME* genes displayed distinct temporal induction patterns, with fold-change increases ranging from 9.14 to 50.90 relative to the control. *ZmME13* showed the highest level of expression at 50.90-fold induction. There was no significant difference in expression after stress at the 1 h time point. However, at the 3 h time point, the expression levels significantly deviated from those observed at the 0 h condition (*p* < 0.05), and this deviation became highly significant by the 6 h to 24 h time points compared to the control (*p* < 0.01). These results indicate that *ZmME* genes are strongly induced in response to drought stress.

#### 2.6.2. High-Temperature Stress at 42 °C

**Figure 6:**
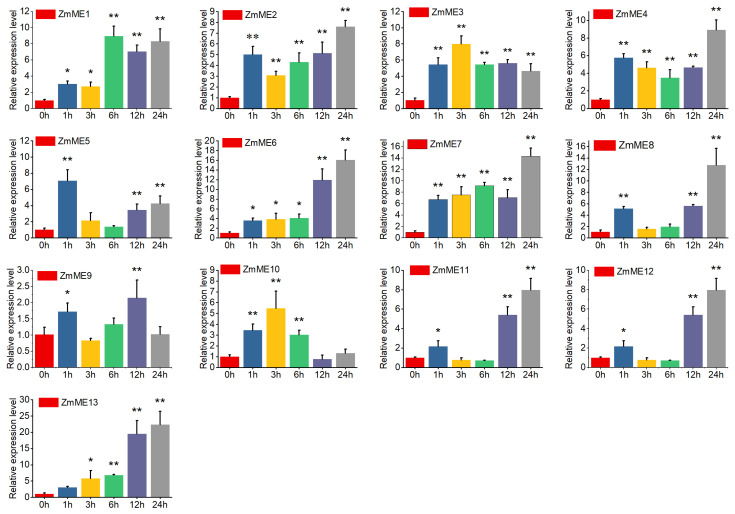
Induced expression results of *ZmME* under high-temperature stress conditions. (*) indicates a significant difference; (**) denotes a highly significant difference. Red represents 0 h (ck), blue represents the expression level at 1 h, yellow at 3 h, green at 6 h, purple at 12 h, and grey at 24 h.

Under 42 °C elevated temperature stress, the expression levels of *ZmME1-13* genes increased from 7.07- to 57.49-fold compared to the control (Figure 6, Table S2). *ZmME13* exhibited the highest level of expression at a 57.49-fold induction. There was no significant difference in expression after stress at the 1 h time point. However, at the 3 h time point, the expression levels significantly deviated from those observed at the 0 h condition (*p* < 0.05), and this deviation became highly significant by the 6 h–24 h time points compared to the control (*p* < 0.01). These results suggest that *ZmME* genes are strongly induced in response to elevated temperature stress.

#### 2.6.3. 4 °C Low-Temperature Stress Treatment

**Figure 7:**
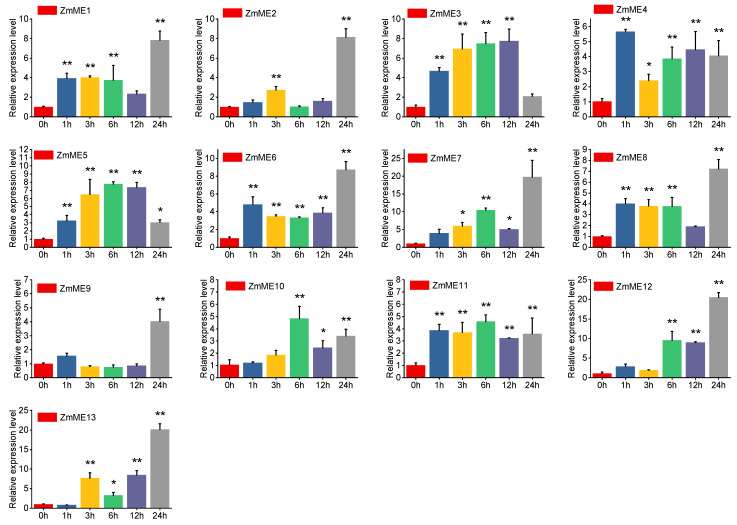
Induced expression results of *ZmME* under low-temperature stress conditions. (*) indicates a significant difference; (**) denotes a highly significant difference. Red represents 0 h (ck), blue represents the expression level at 1 h, yellow at 3 h, green at 6 h, purple at 12 h, and grey at 24 h.

Under 4 °C cold stress treatment, the expression levels of *ZmME1-13* increased from 7.98-fold to 45.19-fold compared to the control (Figure 7, Table S3). The highest fold increase was observed in *ZmME7*, reaching up to 45.19-fold relative to the control. No significant changes were observed after stress at the 1 h time point. However, there were significant differences between expression levels at 3 h and 12 h and those at 0 h (*p* < 0.05). At 6 h and 24 h, highly significant differences were observed in expression levels compared to the control (*p* < 0.01). Based on these findings, it can be inferred that *ZmME* genes are involved in the cold response of seedling maize plants.

#### 2.6.4. 20% PEG Stress Treatment

**Figure 8:**
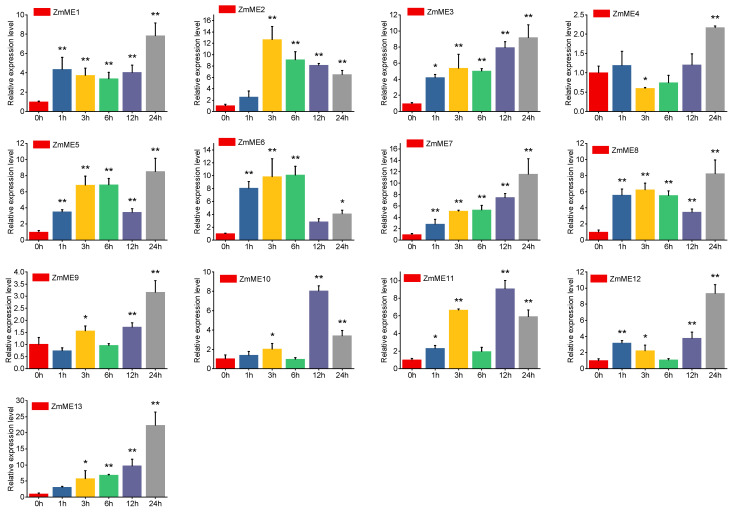
Induced expression results of *ZmME* PEG under stress conditions. (*) indicates a significant difference; (**) denotes a highly significant difference. Red represents 0 h (ck), blue represents the expression level at 1 h, yellow at 3 h, green at 6 h, purple at 12 h, and grey at 24 h.

Under the 20% PEG treatment, the significant induction of *ZmME* genes was observed. The expression levels of *ZmME1-13* were, respectively, increased to 5.93-fold and up to 47.75-fold compared to the control (Figure 8, Table S4). Among these, *ZmME13* exhibited the highest level of expression, reaching 47.75-fold relative to the control. No significant changes were observed after treatment at the 1 h time point. However, a significant difference was detected between expression levels at 3 h and those at 0 h (*p* < 0.05). Furthermore, highly significant differences were observed in expression levels between the 6 h to 24 h treatments and the control (*p* < 0.01). These findings suggest that *ZmME* is also induced by PEG stress and shows a time-dependent pattern of expression.

#### 2.6.5. 250 mmol·L^−1^ NaCl Stress Treatments

**Figure 9:**
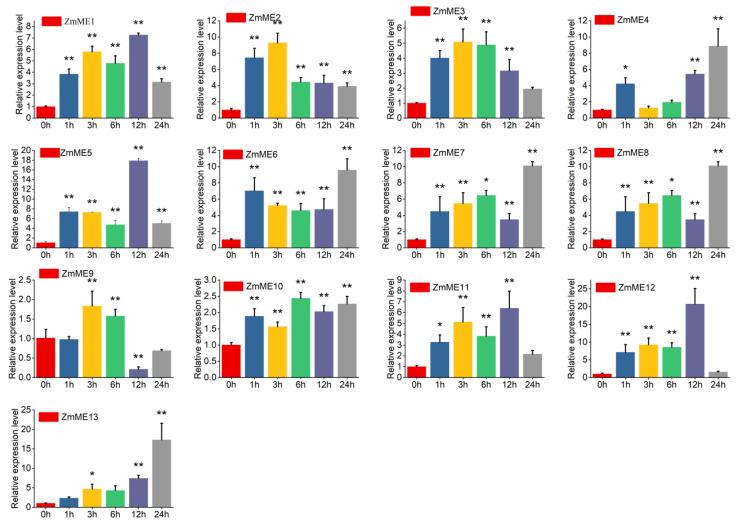
Induced expression results under *ZmME* NACL stress conditions. (*) indicates a significant difference; (**) denotes a highly significant difference. Red represents 0 h (ck), blue represents the expression level at 1 h, yellow at 3 h, green at 6 h, purple at 12 h, and grey at 24 h.

After treatment with 250 mmol·L^−1^ NaCl, the expression levels of *ZmME* ranged from 5.29-fold to 47.20-fold compared to the control (Figure 9, Table S5). Among these, *ZmME13* exhibited the highest expression level at various time points: up to 47.20-fold relative to the control after treatment. For all other members in this group, their expression levels progressively declined over time, with significant differences observed between expression values at different time points and those measured at 0 h (*p* < 0.01). Notably, *ZmME12* exhibited the highest level of expression among these genes across the entire stress period. After exposure to high salt stress for 24 h, its expression level significantly decreased back to baseline levels comparable to the untreated samples (*p* < 0.01). These findings suggest that *ZmME* is also induced by high salt stress and undergoes a dynamic pattern of expression over different time periods under such treatment conditions.

## 3. Discussion

ME is widely distributed in animals, plants, and microorganisms [28,29]. As a key enzyme participating in the tricarboxylic acid (TCA) cycle, it plays essential roles in organismal metabolism. In plants, the malic enzyme is involved in multiple critical metabolic pathways, including photosynthesis, respiration, and lipid biosynthesis [30]. It contributes significantly to maintaining cellular osmotic potential, stabilizing cytoplasmic pH, and regulating ion uptake balance in plant roots, making it one of the most vital enzymes in plant life activities. Therefore, investigating the identification of the *ZmME* gene family and its gene expression under stress conditions is crucial for providing important genetic resources for drought-resistant maize breeding. In this study, we successfully identified 13 *ZmME* gene family members using bioinformatics approaches based on known protein sequences of six Arabidopsis *ME* genes. Beyond maize, the *ME* gene family has been characterized in other species. According to the latest reports from NCBI’s Genome Data Viewer, rice contains 28 *ME* genes, whereas wheat harbors 39 *ME* genes, and they can have the same or different responses to different stresses. This may be related to the physicochemical properties of *ME* genes and the fact that each member assumes a different function in metabolism [31,32]. Chromosomal localization and covariance studies revealed that most of the genes were located on chromosomes, and the distribution of the chromosomes was shown to be uneven. The *ZmME* gene was present on only eight chromosomes and was unevenly distributed, but no significant correlation was obtained between the chromosome length and the number, while some of the genes were concentrated at either top or bottom of the chromosome. In the *ME* gene family, distinct chromosomal localization patterns were observed across species. Four *NADP-ME* isoforms designated *MeNADP-ME1* to *MeNADP-ME4* were identified in cassava Manihot esculenta, with *MeNADP-ME1* and *MeNADP-ME2* mapped to chromosome 16, while *MeNADP-ME3* and *MeNADP-ME4* were localized to chromosomes 11 and 4, respectively [33]. Similarly, pepper Capsicum annuum harbors five *NADP-ME* genes, *CaNADP-ME1* to *CaNADP-ME5*, distributed across chromosomes 3, 5, 8, 9, and 12 [34]. This differential chromosomal distribution of *ME* family members in both species suggests potential functional diversification through sub functionalization or neofunctionalization, possibly reflecting distinct regulatory mechanisms and synergistic roles in plant metabolic processes.

Phylogenetic analysis of the *ZmME* gene family revealed eight sub-families. While the first subfamily contained three *ZmME* genes, the remaining subfamilies each harbored one to two *ZmME* genes. Notably, phylogenetic analysis revealed that malic enzyme genes from maize exhibit the closest evolutionary relationship with their orthologs in teosinte, suggesting conserved functional roles in these closely related species. Thirty-five plant NADP-MEs were classed into four distinct groups, forming the resulting phylogenetic tree: Group I consists of dicot cytosolic proteins, Group II consists of monocots, Group III encompasses dicot plastidic NADP-MEs, and Group IV includes dicot and monocot cytosolic proteins. (Fu, Zhen-Yan et al.). *ZmcytME*, *C4(3)-NADP-ME* and *OscytME3* are classified as Group IV. The discrepancy with the other monocots *NADP-ME* indicates there are two independent evolution branches of *NADP-ME* in monocots. One branch represents the evolution of C4 cytosolic isoforms and the other represents the evolution of plastidic isoforms [35].

Through comprehensive analysis of conserved motifs, structural domains, and gene architectures of *ZmME* family members, we ultimately classified the 13 *ZmME* gene members into four subfamilies. The investigation of the conserved motifs revealed that ME proteins from identical subfamilies share most identical motifs while certain motifs exhibited specific spatial distributions restricted to identical motifs. In Arabidopsis, *AtNAD-ME1* and *AtNAD-ME2* were found to have 19 exons, and *AtNAD-ME3* and *AtNAD-ME4* have 18 exons [36]. The presence and distribution of exons and introns also play key roles in the evolution of gene families [37]. In this study, among the 13 *ZmME* gene, *ZmME5* has only one motif, while the remaining genes contain multiple motifs, possibly due to mutations or duplication of conserved domains during evolution, which may enhance its binding affinity to downstream genes. We inferred that member within the same subfamily consistently shared identical conserved motif compositions, suggesting potential functional overlap and complementarity.

Cis-acting elements are critical regulatory components governing gene expression, modulating responses to developmental processes and environmental stimuli [38]. Promoter regions of *ZmME* family genes harbor diverse functional elements, including core promoter motifs (e.g., CAAT-box and TATA-box), as well as hormone- and stress-responsive elements such as ABA-responsive elements, defense/stress-responsive elements, heat- and cold-responsive elements. Similarly, hormone-responsive elements and ABA-responsive elements were found in bell peppers. Nevertheless, ABRE (ACGT-containing abscisic acid response element), which is involved in abscisic acid (ABA) responsiveness, was the cis-regulatory element that exerted the most remarkable positive effect on *CaNADP-ME4* [34]. Additionally, abiotic stress- and light-responsive elements are widely distributed in *ZmME* promoters. Previous studies have demonstrated a significant positive correlation between upstream promoter regions and their associated cis-acting elements [39,40,41], indicating that *ZmME* gene family members likely participate in light signaling, stress adaptation, and hormonal regulation. Notably, as Poaceae members, both maize and rice exhibit enriched light-responsive elements in *ZmME* promoter regions, suggesting these elements play pivotal roles in maize growth and non-stress physiological processes. Intriguingly, distinct combinatorial patterns of cis-acting elements among genes—such as *ZmME5* and *ZmME13*—may reflect functional specialization. For instance, genes containing “anaerobic induction elements” might be activated under hypoxic conditions (e.g., waterlogging), while those with “endosperm expression elements” could regulate seed development or storage compound biosynthesis. *ZmME13* harbors drought-inducible MYB-binding sites in its promoter, which likely drives its highly induced expression under drought stress. These findings collectively underscore the functional importance of *ZmME* genes in plant growth and environmental adaptation.

Expression pattern analysis provides a crucial foundation for determining gene functions [42] in the *ZmME* gene under different conditions of stress treatment. Plants possess a sophisticated stress signal transduction system that activates their intrinsic defense mechanisms through hierarchical signaling cascades when subjected to external stresses. Most stress signals can induce the expression of defense-related genes [43]. For instance, expression profiling of the *WRKY* gene family in maize has revealed its expression patterns under abiotic stresses, offering new insights into the signaling pathways involved in maize responses to abiotic stress [44]. Overexpression of *NADP-ME* in rice enhances the salt tolerance of transgenic Arabidopsis seedlings. Moreover, the NADP-ME activity in leaves and roots also increases with the increase in the NaCl concentration. Similarly, the activity of NAD-ME and NADP-ME in the leaves of eucalyptus increased under salt stress, which provided a reducing power for the cells to resist salt stress. We speculate that *ME* is mainly used in three ways after being induced by salt stress. The enhanced activity of NADP-ME confers salt stress tolerance through three distinct mechanisms: First, it elevates NADPH production, which scavenges reactive oxygen species (ROS) to mitigate oxidative damage, second, the generated NADP serves as a cofactor for osmo-protectant biosynthesis, while the concomitant accumulation of malate the enzymatic product of NADP-ME paradoxically exacerbates osmotic stress under saline conditions. Finally, this malate synthesis pathway contributes to cytosolic pH homeostasis by counteracting the alkalinization induced by salt stress [17]. Northern and Western blot data indicated that in aloe, the *NADP-ME* gene was strongly induced by high salt. The increases in transcript levels and translation levels of *NADP-ME* in leaf tissue during salt stress suggest that this gene is responsible in part for salt tolerance in aloe [45]. Studies have shown that long-term low temperature induces an increase in the malate content or NADP-ME activity in rye (*Secale cereale* L.). When the low-temperature stress is removed, both the malic acid content and enzyme activity decrease. The above results indicate that rye not only regulates the malic acid content as a good osmotic adjustment substance but also responds to the osmotic stress caused by low temperature by regulating the NADP-ME activity. NADP-ME can not only be a good osmotic adjustment substance by participating in some enzymatic reactions but also balance the higher solute concentration in cells caused by low temperature [46]. ME is not only related to low-temperature stress, but also plays an important role in high-temperature stress. The NAD-ME activity in C3 plant rice increased with increasing temperature within a certain temperature range. Under drought stress, a new ~122 kDa NAD-ME subtype was formed in the mitochondria of the bundled sheath (BS) cells of the amaranth. After watering was resumed, the isomer (~122 kDa) disappeared again. Due to the emergence of new isomers, the activity of *NAD-ME* in the mitochondria of BS cells increased, which seems to indicate that the new *NAD-ME* subtype is related to plant drought resistance [47]. However, its specific mechanism of action requires further research. Similarly, using quantitative RT-PCR and immunochemical methods, the specific activity of NADP-ME in tobacco leaves was determined to significantly increase under drought stress, and the de novo synthesis of NADP-ME was enhanced. During the recovery period, the activity of the test enzyme recovered to near its basal level [16]. Changes in enzyme activity and content provide indirect evidence for the involvement of NADP-ME in the response to drought stress. The above results were consistent with the findings of this work, in which *ME* genes were expressed and played different roles to different degrees under stress treatments such as drought, low temperature, high temperature, and salt.

## 4. Materials and Methods

### 4.1. Experimental Methods

#### 4.1.1. Identification of ZmME Gene Family Members

For the protein sequence information of six MEs (NADP-ME and NAD-ME) reported by the uniport database, https://www.uniprot.org/, for maize, teosinte, sorghum, rice, barley, millet, and proso millet, we downloaded their genome and annotation files. We used the local BLASTP v.2.6.0 (E < 1 × 10^−10^, identify > 50%) to perform sequence comparison analysis, with the results being the candidate members of the *ME* gene family in maize. We, reassigned the names of the members of this gene family based on their locations on the chromosomes. We used online software ExPASy, http://web.expasy.org/protparam/ (accessed on 18 December 2024) to predict and analyze the basic physicochemical properties of the *ME* proteins formally, including the molecular weight, amino acid number, and isoelectric point.

#### 4.1.2. Chromosomal Localization and Collinearity Analysis

Maize and sorghum genome-wide co-linearity analysis was conducted using McScan X with a minimum module gene number of 5. *ME* genes from maize and sorghum were aligned, and orthologous genes (>90% identity) were identified as originating from the same source. A co-linearity map was generated for maize and sorghum, with red highlighting applied to *ME* genes on different chromosomes that belonged to the same source.

#### 4.1.3. Phylogenetic Analysis of Gene Families

The amino acid sequences of the identified gene family members were aligned using Clustal W for multiple sequence alignment. Phylogenetic trees were subsequently constructed with MEGA11 software, employing the Neighbor-Joining method with 1000 bootstrap replicates and default parameters. This approach refined the tree topology, allowing clear visualization of distinct clades and their branching characteristics [48].

#### 4.1.4. Conserved Motifs, Structural Domains and Gene Structure Analysis of Gene Family Members

Structural information of the *ZmME* family genes was obtained from the genome data of maize. The conservative base prediction of the *ZmME* protein sequences was performed using the online analysis software MEME (http://meme-suite.org/tools/meme (accessed on 18 December 2024)) with parameter settings as follows: maximum number of motifs as 10, occurrences of a single motif, set to zero or one per sequence [40]. The remaining parameters were set to default. Gene structure information was extracted from the genome annotation files. TBtools v0.665 were utilized to integrate and visualize the motif, conservative protein structure, and gene structural results of the *ZmME* family [41].

#### 4.1.5. Gene Family Promoter Cis-Acting Element Analysis

We used seqkit to extract the upstream 2000 bp sequences of the transcription start sites (TSSs) for the *ZmME* family genes. These sequences were then submitted to the Plant CARE website http://bioinformatics.psb.ugent.be/webtools/plantcare/html/ (accessed on 18 December 2024) for functional prediction, aiming to identify potential upstream cis elements involved in gene regulation [42]. The prediction results were classified and organized before being integrated into a comprehensive visualization using TBtools based on motif conservation analysis and genomic structural data of the *ZmME* family genes.

#### 4.1.6. ZmME Gene Family Expression Analysis

We selected size-equivalent B73 maize inbreeding lines as the experimental material. The seeds were soaked in a 0.5% hypochlorite sodium solution, stirred and soaked again, and then washed repeatedly with sterile water to ensure cleanliness. The planting was carried out in a controlled environment with an ambient temperature of 25 °C and relative humidity of 60%. After germination for seven days, the seedlings were transplanted into plastic pots. At the stage of three leaves and one stem, different stress treatments were applied: simulated drought stressed conditions (with air filters set at 50% relative humidity), high-temperature stressed conditions (42 °C), low-temperature stressed conditions (−4 °C), high NaCl stressed conditions (250 mmol/L), and PEG6000 polyethylene glycol stressed conditions. Sampling was performed at different time points: 0 h, 1 h, 3 h, 6 h, 12 h, and 24 h after the stress treatments. The samples were collected with liquid nitrogen, divided into sealed bags with labels, and stored in a −80 °C freezer for future RNA extraction and cDNA synthesis.

#### 4.1.7. Maize ZmME Gene qRT-PCR Validation

qRT-PCR using *ZmActin-1* as the reference gene was performed for the analysis of the relative expression levels of the *ZmME* gene family. The reaction mixture contained 10 μL of TB Green Premix Ex Taq II (Tli RNaseH Plus), 0.8 μL each of PCR forward primer and PCR reverse primer, 2 μL DNA template, and 6.4 μL of sterile water, totaling a final volume of 20 μL. The relative expression levels were calculated based on the experimental results obtained using the TB Green Premix Ex Taq II (Tli RNaseH Plus) qPCR mix kit (Takara) in the Light Cycler 96 fluorescent quantitative PCR instrument (Roche).

#### 4.1.8. Data Processing

Data processing was performed using SPSS 20.0 software for statistical analysis and analysis of variance (ANOVA), with the mean values and standard deviations calculated (significant differences were defined at *p* < 0.05, and highly significant differences at *p* < 0.01).

## 5. Conclusions

This study identified 13 members of the *ZmME* gene family in maize, and conducted a comprehensive analysis of their physicochemical properties, evolutionary relationships, and collinearity relationship. The *ZmME* gene family exhibits high stability during long-term evolution and high conservation among crop legumes. Through the analysis of promoter-sequential elements, it was discovered that the *ZmME* family contains stress-responsive motifs and drought-induction binding sites. Under drought stress, these genes may play a regulatory role. Notably, based on the highly induced expression of *ZmME13* under various stress treatments, we conclude that *ZmME13* is a promising candidate gene with significant potential for stress resistance. Currently, *ME* gene families from many other species remain understudied, and the functional roles of their members and their involvement in stress response expression patterns are still unclear. Therefore, this study provides a theoretical foundation for subsequent investigations into the molecular biology functions of *ZmME* gene family members in maize.

## Figures and Tables

**Table 1:** Genome-wide identification and characterization of *ZmME*.

Gene Name	Chromosomes	Start	End	Gene Length	Number of Amino Adds	Molecular Weight	Theoretical Pl	CDS (bp)	Grand Average of Hydropathicity (GRAVY)
*Zm ME1*	chr1	47,534,292	47,535,943	1652	189	21,681.92	9.2	567	−0.324
*Zm ME2*	chr1	290,259,636	290,260,669	1034	204	23,001.29	5.22	612	−0.361
*Zm ME3*	chr3	7,184,482	7,190,135	5654	636	69,818.85	6.2	1908	−0.168
*Zm ME4*	chr3	130,704,894	130,717,659	12766	138	15,622.11	5.56	414	0.291
*Zm ME5*	chr3	164,884,990	164,886,965	1976	178	19,890.73	8.87	534	−0.33
*Zm ME6*	chr3	206,946,472	206,951,192	4721	480	52,749.47	5.93	1440	−0.081
*Zm ME7*	chr4	2,155,524	21,560,705	5182	240	27,447.67	7.07	720	−0.248
*Zm ME8*	chr5	24,840,267	24,847,307	7041	618	67,809.04	8	1854	−0.121
*Zm ME9*	chr6	141,031,530	141,035,585	4056	570	62,893.99	5.69	1710	−0.147
*Zm ME10*	chr6	150,321,630	150,375,807	54178	644	70,694.85	6.35	1932	−0.144
*Zm ME11*	chr7	159,213,544	159,223,682	10139	619	69,133.99	6.09	1857	−0.251
*Zm ME12*	chr8	110,806,909	110,810,557	3649	570	62,703.59	5.74	1710	−0.154
*Zm ME13*	chr8	181,572,829	181,578,195	5367	593	64,958.31	5.82	1779	−0.092

**Table 2:** Primer information used in the transcriptional expression level detection of *ZmME*genes.

Primer Name	Forward Primer Sequence (5′-3′)	Reverse Primer Sequence (5′-3′)
ZmME1	ACCTCTTCCTCTCGAGGCTT	ACTGAAGGCATTAGGCCACC
ZmME2	GCTTGCTTGCCGATCACATT	GGGGTCAGAAGCTTCCAGTT
ZmME3	GGACCTACAGGAACACCCTG	GACCCTTGTTGTGGTGTGGA
ZmME4	AACGTGTGACTGTCGAGGAG	GCATTGCAGGACCCTATCCA
ZmME5	ATGGTGCAGGCTCTAGCAAA	GAGGCCACAATCAGTCCCTC
ZmME6	CTGGAGCTATCCGCGTTCAT	GGGGCTGTACATGCAACTCT
ZmME7	CCTATGGCGACAACTTCGGT	CGTCTCCCCCTCCTTAGTGT
ZmME8	TGGGATTGGTTTAGGTGCCC	CTCAGCCAGGTCTTCAGCAA
ZmME9	AGGAGCATCCAGGTCATCGT	ACGGCGGTCATGAACTCTTCAA
ZmME10	TGCTTCCTCCTGCAGTTGTT	AGGACCTTCCCTTTGTCCCT
ZmME11	ACAAGAGCACCGTCTTGAGG	ACTCCAATACCAGCACTGCC
ZmME12	TTCCCGTTGGCAAGCTCTC	GTAGTTTTGCTTGACGGCGGC
ZmME13	ATTTGGCAGTGGAAGCCCAT	CATAAGCTTTTGCGGCGACA
ZmActin-1	TGAAACCTTCGAATGCCCAG	GATTGGAACCTGGTGGCTCA

## Data Availability

Data from this study can be found in the article and Supplementary Materials.

## Supplementary Materials

The following supporting information can be downloaded at https://www.mdpi.com/article/10.3390/plants14111603/s1, Table S1: Induced expression results of *ZmME* under drought stress conditions; Table S2: Induced expression results of *ZmME* under high-temperature stress conditions; Table S3: Induced expression results of *ZmME* under low-temperature stress conditions; Table S4: Induced expression results of *ZmME* PEG under stress conditions; Table S5: Induced expression results under *ZmME* NACL stress conditions.

## References

[1] Aida M., Ishida T., Fukaki H., Fujisawa H., Tasaka M. (1997). Genes involved in organ separation in arabidopsis: An analysis of the cup-shaped cotyledon mutant. Plant Cell.

[2] Kopecka R., Kameniarova M., Cerny M., Brzobohaty B., Novak J. (2023). Abiotic stress in crop production. Int. J. Mol. Sci..

[3] Ribaut J.M., Hoisington D.A., Deutsch J.A., Jiang C., Gonzalez-de-Leon D. (1996). Identification of quantitative trait loci under drought conditions in tropical maize. 1. Flowering parameters and the anthesis-silking interval. Theor. Appl. Genet..

[4] Liang Y., Kang K., Gan L., Ning S., Xiong J., Song S., Xi L., Lai S., Yin Y., Gu J. (2019). Drought-responsive genes, late embryogenesis abundant group3 (LEA3) and vicinal oxygen chelate, function in lipid accumulation in brassica napus and arabidopsis mainly via enhancing photosynthetic efficiency and reducing ROS. Plant Biotechnol. J..

[5] Lobell D.B., Roberts M.J., Schlenker W., Braun N., Little B.B., Rejesus R.M., Hammer G.L. (2014). Greater sensitivity to drought accompanies maize yield increase in the U.S. Midwest. Science.

[6] Li Y., Guan K., Schnitkey G.D., DeLucia E., Peng B. (2019). Excessive rainfall leads to maize yield loss of a comparable magnitude to extreme drought in the United States. Glob. Change Biol..

[7] Kim K.H., Lee B.M. (2023). Effects of climatechange and drought tolerance onmaize growth. Plants.

[8] Zandalinas S.I., Mittler R., Balfagón D., Arbona V., Gómez-Cadenas A. (2018). Plant adaptations to the combination of drought and high temperatures. Physiol. Plant.

[9] Nykiel M., Gietler M., Fidler J., Prabucka B., Rybarczyk-Płońska A., Graska J., Boguszewska-Mańkowska D., Muszyńska E., Morkunas I., Labudda M. (2022). Signal transduction incereal plants struggling with environmental stresses: From perception to Response. Plants.

[10] Voll L.M., Zell M.B., Engelsdorf T., Saur A., Wheeler M.G., Drincovich M.F., Weber A.P., Maurino V.G. (2012). Loss of cytosolic NADP-malic enzyme 2 in arabidopsis thaliana is associated with enhanced susceptibility tocolletotrichum higginsianum. New Phytol..

[11] Maurino V.G., Saigo M., Andreo C.S., Drincovich M.F. (2001). Non-photosynthetic ‘malic enzyme’ from maize: A constituvely expressed enzyme that responds to plant defence inducers. Plant Mol. Biol..

[12] Guo H.P., Chen H.M., Hong C.T., Jiang D., Zheng B.S. (2017). Exogenous malic acid alleviates cadmium toxicity in miscanthus sacchariflorus through enhancing photosynthetic capacity and restraining ROS accumulation. Ecotoxicol. Environ. Saf..

[13] Wang P., Lu S.X., Cao X.J., Ma Z.H., Chen B.H., Mao J. (2023). Physiological and transcriptome analyses of the effects of excessive water deficit on malic acid accumulation in apple. Tree Physiol..

[14] Katayama N., Iwazumi K., Suzuki H., Osanai T., Ito S. (2022). Malic enzyme, not malatedehydrogenase, mainly oxidizes malate that originates from the tricarboxylic acid cycle in cyanobacteria. mBio.

[15] Maier A., Zell M.B., Maurino V.G. (2011). Malate decarboxylases: Evolution and roles of NAD(P)-ME isoforms in species performing C_4_ and C_3_ photosynthesis. J. Exp. Bot..

[16] Doubnerová Hýsková V., Miedzińska L., Dobrá J., Vankova R., Ryšlavá H. (2014). Phosphoenolpyruvate carboxylase, NADP-malic enzyme, and pyruvate, phosphate dikinase are involved in the acclimation of *Nicotiana tabacum* L. to drought stress. J. Plant Physiol..

[17] Sun X., Han G.L., Meng Z., Lin L., Sui N. (2019). Roles of malic enzymes in plant development and stress responses. Plant Signal Behav..

[18] Chen Q.Q., Wang B.P., Ding H.Y., Zhang J., Li S.C. (2019). Review: The role of NADP-malic enzyme in plants under stress. Plant Sci..

[19] Cushman J.C. (1992). Characterization and expression of a NADP-malic enzyme cDNA induced by salt stress from the facultative crassulacean acid metabolism plant, Mesembryanthemum crystallinum. Eur. J. Biochem..

[20] Holtum J.A., Winter K. (1982). Activity of enzymes of carbon metabolism during the induction of Crassulacean acid metabolism in *Mesembryanthemum crystallinum* L.. Planta.

[21] Li X.F., Zhang X.X., Takio N., Liu C.K. (2012). Expression characteristics of Rice (*Oryza sativa* L.) malic enzyme (OsNADP-ME3) gene under environmental stress. Genom. Appl. Biol..

[22] Cheng Y.X., Long M. (2007). A cytosolic NADP-malic enzyme gene from rice (*Oryza sativa* L.) confers salt tolerance in transgenic Arabidopsis. Biotechnol. Lett..

[23] Shao H.B., Liu Z.H., Zhang Z.B., Chen Q.J., Chu L.Y., Brestic M. (2011). Biological roles of crop NADP malic enzymes and molecular mechanisms involved in abiotic stress. Afr. J. Biotechnol..

[24] Wen Z.B., Zhang M.L. (2017). Possible involvement of phosphoenolpyruvate carboxylase and NAD-malic enzyme in response to drought stress. A case study: A succulent nature of the C_4_-NAD-ME type desert plant, *Salsola lanata* (Chenopodiaceae). Funct. Plant Biol..

[25] Abbas K., Li J.R., Gong B.B., Lu Y.S., Wu X.L., Lü G.Y., Gao H.B. (2023). Drought stress tolerance in vegetables: The functional role of structural features, key gene pathways, and exogenous hormones. Int. J. Mol. Sci..

[26] Xu W., Yan Q. (2003). Advances in the research of cold resistance in sugarcane. Sugarcane.

[27] Liu Z.H., Zhang Z.B., Chu L.Y., Shao H.B. (2010). The corresponding relationship between roles of NADP-malic enzymes and abiotic stress in plants. Emir. J. Food Arg..

[28] Zou Y., Zhang Z.X., Zeng Y.J., Hu H.Y., Hao Y.J., Huang S., Li B. (2024). Common methods for phylogenetic tree construction and their implementation in R. Bioengineering.

[29] Frenkel R. (1975). Regulation and physiological functions of malic enzymes. Curr. Top. Cell Regul..

[30] Nei M. (1996). Phylogenetic analysis in molecular evolutionary genetics. Annu. Rev. Genet..

[31] Peng X.J., Zhao Y.Z., Li X.M., Wu M., Chai W.B., Sheng L., Wang Y., Dong Q., Jiang H.Y., Cheng B.J. (2015). Genomewide identification, classification and analysis of NAC type gene family in maize. J. Genet..

[32] Xu J.H., Messing J. (2008). Organization of the prolamin gene family provides insight into the evolution of the maize genome and gene duplications in grass species. Proc. Natl. Acad. Sci. USA.

[33] Li H.Z., Xiao J., Chen J.H., Shen X., Luo J., Guo F.G., Wang S.F., Xu L.Y., Guo X., Wang S. (2025). Identification of the cassava NADP-ME gene family and its response and regulation in photosynthesis. Front. Plant Sci..

[34] Taboada J., González-Gordo S., Muñoz-Vargas M.A., Palma J.M., Corpas F.J. (2023). NADP-dependent malic enzyme genes in sweet pepper fruits: Involvement in ripening and modulation by nitric oxide (NO). Plants.

[35] Fu Z.Y., Zhang Z.B., Hu X.J., Shao H.B., Ping X. (2009). Cloning, identification, expression analysis and phylogenetic relevance of two NADP-dependent malic enzyme genes from hexaploid wheat. Comptes Rendus Biol..

[36] Wheeler M.C., Tronconi M.A., Drincovich M.F., Andreo C.S., Flügge U.I., Maurino V.G. (2005). A comprehensive analysis of the NADP-malic enzyme gene family of Arabidopsis. Plant Physiol..

[37] Xu G.X., Guo C.C., Shan H.Y., Kong H.Z. (2012). Divergence of duplicate genes in exon-intron structure. Proc. Natl. Acad. Sci. USA.

[38] Chow C.N., Chiang-Hsieh Y.F., Chien C.H., Zheng H.Q., Lee T.Y., Wu N.Y., Tseng K.C., Hou P.F., Chang W.C. (2018). Delineation of condition specific *Cis*- and *Trans*-acting elements in plant promoters under various Endo- and exogenous stimuli. BMC Genom..

[39] Hernandez-Garcia C.M., Finer J.J. (2014). Identification and validation of promoters and cis-acting regulatory elements. Plant Sci..

[40] Girin T., Lejay L., Wirth J., Widiez T., Palenchar P.M., Nazoa P., Touraine B., Gojon A., Lepetit M. (2007). Identification of a 150 bp cis-acting element of the AtNRT2.1 promoter involved in the regulation of gene expression by the N and C status of the plant. Plant Cell Environ..

[41] Li R.X., Zhu F.D., Duan D. (2020). Function analysis and stress-mediated cis-element identification in the promoter region of VqMYB15. Plant Signal Behav..

[42] Yue C., Cao H.L., Lin H.Z., Hu J., Ye Y.J., Li J.M., Hao Z.L., Hao X.Y., Sun Y., Yang Y.J. (2019). Expression patterns of alpha-amylase and beta-amylase genes provide insights into the molecular mechanisms underlying the responses of tea plants (*Camellia sinensis*) to stress and postharvest processing treatments. Planta.

[43] Jwa N.S., Agrawal G.K., Tamogami S., Yonekura M., Han O., Iwahashi H., Rakwal R. (2006). Role of defense/stress-related marker genes, proteins and secondary metabolites in defining rice self-defense mechanisms. Plant Physiol. Biochem..

[44] Hu W.J., Ren Q.Y., Chen Y.L., Xu G.L., Qian Y.X. (2021). Genome-wide identification and analysis of WRKY gene family in maize provide insights into regulatory network in response to abiotic stresses. BMC Plant Biol..

[45] Sun S.B., Shen Q.R., Wan J.M., Liu Z.P. (2003). Induced expression of the gene for NADP-malic enzyme in leaves of *Aloe vera* L. under salt stress. Acta Biochim. Biophys. Sin..

[46] Crecelius F., Streb P., Feierabend J. (2003). Malate metabolism and reactions of oxidoreduction in cold-hardened winter rye (*Secale cereale* L.) leaves. J. Exp. Bot..

[47] Babayev H., Mehvaliyeva U., Aliyeva M., Feyziyev Y., Guliyev N. (2014). The study of NAD-malic enzyme in *Amaranthus cruentus* L. under drought. Plant Physiol. Biochem..

[48] Farris J.S., Albert V.A., Källersjö M., Lipscomb D., Kluge A.G. (1996). Parsimony jackknifing outperforms neighbor-joining. Cladistics.

